# Screening for Primordial RNA–Peptide Interactions Using High-Density Peptide Arrays

**DOI:** 10.3390/life13030796

**Published:** 2023-03-15

**Authors:** Felix Jenne, Ivan Berezkin, Frank Tempel, Dimitry Schmidt, Roman Popov, Alexander Nesterov-Mueller

**Affiliations:** 1 Institute of Microstructure Technology, Karlsruhe Institute of Technology, DE-76344 Eggenstein-Leopoldshafen, Germany; 2 Axxelera UG, DE-76189 Karlsruhe, Germany

**Keywords:** RNA peptide interactions, high-density peptide arrays, the origin of the genetic code

## Abstract

RNA–peptide interactions are an important factor in the origin of the modern mechanism of translation and the genetic code. Despite great progress in the bioinformatics of RNA–peptide interactions due to the rapid growth in the number of known RNA–protein complexes, there is no comprehensive experimental method to take into account the influence of individual amino acids on non-covalent RNA–peptide bonds. First, we designed the combinatorial libraries of primordial peptides according to the combinatorial fusion rules based on Watson–Crick mutations. Next, we used high-density peptide arrays to investigate the interaction of primordial peptides with their cognate homo-oligonucleotides. We calculated the interaction scores of individual peptide fragments and evaluated the influence of the peptide length and its composition on the strength of RNA binding. The analysis shows that the amino acids phenylalanine, tyrosine, and proline contribute significantly to the strong binding between peptides and homo-oligonucleotides, while the sum charge of the peptide does not have a significant effect. We discuss the physicochemical implications of the combinatorial fusion cascade, a hypothesis that follows from the amino acid partition used in the work.

## 1. Introduction

The uniqueness of ribosomal translation [1] points to its complex evolutionary development [2] which is likely to have involved the co-evolution of ribonucleic acids and peptides [3]. The recently demonstrated peptide synthesis on complementary short oligonucleotide pairs [4] has become a strong argument in support of the “RNA/peptide world” concept [5,6].

The high-throughput screening technique SELEX [7,8] has been actively used to test the stereochemical hypothesis [9,10,11,12] relating to the origin of the genetic code as a central element of ribosomal translation. The stereochemical hypothesis suggests that the codon assignment in the standard genetic code (SGC) originated from the direct selective binding of amino acids to their cognate codons. Although experimental evidence for the stereochemical hypothesis has not been obtained [13], specific binding of some individual cognate codons to amino acids has been reported (e.g., arginine, isoleucine, and histidine) [12,14,15]. Those studies focused on diverse RNA structures as selective binders to amino acids or their derivatives, while the diversity of amino acid sequences was disregarded.

The rapid increase in the number of experimentally validated RNA-binding proteins over the past decade has enabled a comprehensive statistical analysis of the physicochemical principles of RNA/protein interactions [16]. Within the experimentally validated RNA-binding proteins, 78% of hydrogen bonds involve amino acid side chains, and the remaining 22% involve the protein backbone [17]. This indicated that sequence specificity plays an important role in the protein component.

Research on non-covalent binding between the mRNA and cognate proteins [18,19,20,21] has led to several findings that we will refer to in this work. In particular, strong support has been provided to the direct templating of unstructured proteins from mRNAs in the era before the development of ribosomal decoding [22,23,24]. However, the statistically derived amino acid/nucleotide interaction preferences can be strongly biased toward the stable ribonucleoprotein complexes as a product of the modern ribosomal translation. In this work, we demonstrate a bottom-up approach to studying primordial RNA/peptide interactions with fully combinatorial high-density peptide arrays [25,26,27,28] to evaluate as many potential interactions as possible.

## 2. Materials and Methods

### 2.1. The Partition of the Standard Genetic Code According to the Combinatorial Fusion of Protocodes

Since the number of possible combinations of interacting ribonucleic acids and peptides grows exponentially with their length, we need a rational partition of the RNA/peptide interaction space.

To solve the code uniqueness problem, Vetsigian et al. [29] pointed out the necessity of the existence of competing entities with alternative codes, the number of which was reduced during horizontal gene transfer (HGT). The modeled curve of convergence to the singular genetic code due to the HGT did not have breaks in the first derivative, which suggested the origin of the genetic code by merging the few most stable entities just before the appearance of the SGC. Although the HGT is one of the most important mechanisms in the evolution of prokaryotes [30,31,32], it is difficult to imagine HGT between cells with different translational apparatuses.

Analyzing complementary interactions between messenger RNAs and their cognate proteins, Polyansky and Zagrovic concluded that the early phase in the code’s development was dominated by G- and C-rich codons and was later extended by the inclusion of A and U bases [22]. Trifonov et al. also suggested that the first codons were G- and C-rich, based on a consensus analysis of 40 different criteria [33].

The combinatorial fusion of AU- and GC-protocodes proposed by Nesterov-Mueller and Popov absorbed these ideas of the competing entities and the separated AU and GC early phases [34]. It described with surprising simplicity the codon assignments in the SGC, including the codons for the non-canonical amino acids, the appearance of stop codons, as well some deviations from the SGC in mitochondria [35].

**Figure 1:**
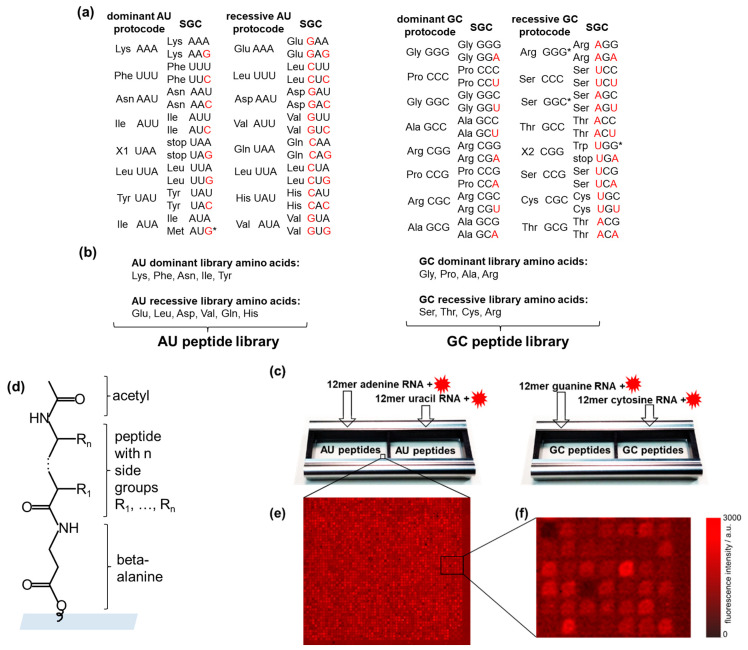
High-throughput screening of RNA–peptide interactions. (**a**) AU- and GC-protocodes and their transformation to the standard genetic code (SGC) after the combinatorial fusion. The codons of the SGC were obtained according to the Watson–Crick mutations A↔G or U↔C in the third position for the dominant protocodes. Watson–Crick mutations for recessive protocodes occurred in the first or the first and third positions. The red letters illustrate these transformations. (**b**) The AU-protocode library consists of two sublibraries: all combinatorial combinations of the amino acids of the dominant protocode (Lys, Phe, Asn, Ile, and Tyr) and all combinatorial combinations of the amino acids of the recessive protocode (Glu, Leu, Asp, Val, Gln, and His). The GC-protocode library consists of two sublibraries: all combinatorial combinations of the amino acids of the dominant protocode (Gly, Pro, Ala, and Arg) and all combinatorial combinations of the amino acids of the recessive protocode (Ser, Thr, Cys, and Arg). (**c**) Location of the peptide libraries in the incubation trays and their incubation with fluorescently labeled 12-mer single-stranded RNA homo-oligomers of adenine, guanine, uracil, and cytosine. (**d**) Peptide structure in the spot: beta-alanine as linker, acetylated N-terminus. (**e**,**f**) Fragments of fluorescence images of peptide chips after incubation with fluorescently labeled RNAs. The pitch size was 60 µm. * The asterisks indicate the codon assignments of the SGC that are discussed in Section 2.2.

Figure 1a shows two dominant and two recessive AU- and GC-protocodes. Note that the amino acids from the dominant and recessive protocodes competed for the same codons. For example, phenylalanine (Phe) competed with leucine (Leu) for the codon UUU, and proline (Pro) competed with serine (Ser) for the codon CCC. The terms “dominant” and “recessive” were taken from classical genetics and refer to the fact that the dominant protocodes did not change their initial codon–amino acid assignments after their combinatorial fusion with the recessive ones. By contrast, the recessive protocodes acquired new triplets.

Combinatorial fusion implies the entry of the other two bases of adenine (A) and uracil (U) into the GC-protocodes, as well as guanine (G) and cytosine (C) into the AU-protocodes. The emergence of new codons in the SGC from the dominant protocodes is described by the well-known Watson–Crick mutations: A↔G or U↔C [36]. These mutations occur in the third position of the codon. The change of codons in the recessive protocodes is subject to a simple “take what’s left” rule: the amino acids occupy the free codons that were left by the dominant protocode so that their original complementarity with another amino acid from the recessive protocode before the fusion is not violated. These are the same mutations A↔G or U↔C, only this time in position one or simultaneously in positions one and three.

### 2.2. The Peptide Libraries of the Protocodes

Assigning the amino acids to the protocodes, we designed the corresponding peptide libraries (Figure 1b). The peptides of the dominant and recessive AU-protocodes consisted of all possible combinations of amino acids Lys, Phe, Asn, Ile, and Tyr, as well as Glu, Leu, Asp, Val, Gln, and His, respectively. The GC peptide library was designed similarly: amino acids Gly, Pro, Ala, and Arg were taken for the dominant GC-protocode, and Ser, Thr, Cys, and Arg for the recessive GC-protocode. We excluded the amino acids Met and Trp from the peptide libraries. According to Trifonov [37], there is a consensus among various approaches that these amino acids entered the code much later than the others. Both amino acids have the lowest frequency in the proteome and, unlike other canonical amino acids, are encoded by only one triplet [38]. Met is encoded by the start codon AUG in SGC, and Trp has taken over the stop codon from the missing amino acid X2. The fact that both Met and Trp are encoded by only one codon could be explained by their late entry into SGC after combinatorial protocode fusion. Jheeta et al. have summarized in detail the reasons for the late entry of Met and Trp into the genetic code [6].

We excluded Leu from the dominant AU-protocode since it could have come into it from a recessive AU code only after the combinatorial fusion of protocodes in place of the previously disappeared amino acid pair X1 and X1* with complementary codons UAA and UUA. Indeed, the deletion of X1 resulted in the formation of two stop codons UAA and UAG, and the codon of X1* was occupied by Leu since it had the same UUA codon in the recessive protocode. The existence of three stop codons in the SGC may be a direct consequence of the disappearance of amino acids X1 and X2 and the late entry of Trp.

We have included Arg in both the dominant and recessive GC-protocodes. However, it should be noted that Arg and Ser should be classified as traveling amino acids, which occupied the GGG and GGS codons in the recessive GC-protocode (Figure 1a). The codon heterogeneity of arginine and serine compared to their other four codons was noticed early and exploited in the development of an extended genetic code by re-assigning the arginine codon AGG to non-canonical amino acids in *Escherichia coli* [39]. The transition of Arg, Ser, and Leu likely occurred after the fusion of protocodes, which provided them with a maximum number of six cognate codons in the SGC.

### 2.3. High-Density Peptide Arrays

Peptide libraries were synthesized as high-density peptide arrays (axxelera UG, Karlsruhe, Germany). The amino acids from the AU-protocodes were presented on 23,403 6-mer peptides and constituted the AU peptide library. Each peptide of the AU peptide library was represented by three copies. The resulting 32,768 fully combinatorial combinations of amino acids from the GC-protocodes were presented as 7-mer peptides. Each peptide of the GS peptide library was represented by two copies. Each library fits in one incubation well (Figure 1c). The peptide spots were allocated randomly on the chip surface to avoid local effects when interacting with RNA. All peptides were N-terminal acetylated to take into account the interactions only due to the side groups (Figure 1d). N-terminal acetylation is a widespread protein modification among eukaryotes and prokaryotes alike [40].

### 2.4. Incubation of the Peptide Chips with RNA

The AU peptide library was incubated with fluorescently labeled 12-mer homo-oligonucleotides of adenine and uracil. The GC peptide library was incubated with fluorescently labeled 12-mer homo-oligonucleotides of guanine and cytosine. We only use homo-oligonucleotides to avoid RNA pairing and secondary structures.

All incubation and washing steps were performed in a ProPlate^®^ Two-Well Chamber (Grace Bio-Labs, Bend, OR, USA), which allowed for a minimized sample incubation volume and the subdivision of the peptide chip (75 mm × 25 mm) into 2 separate incubation wells for each slide. Incubation started with a pre-swelling of the peptide array with PBS-T (1 × phosphate-buffered saline (PBS), Sigma Aldrich, St. Louis, MO, USA pH 7.4, 0.05% *v*/*v* Tween 20, Sigma Aldrich) for 10 min. The RNAs (biomers.net GmbH, Ulm, Germany) with fluorescence dyes Cyanine 5 or Cyanine 3 (5’ modification [41]) were dissolved in PBS-T to a concentration of 50 µg/mL. Note that the interactions were studied under physiological conditions, assuming their existence in primordial entities with the protocodes. PBS is often used to study selective peptide–antibody interactions [42,43]). Two different dyes were used to eliminate the effect of the dye on the strength of RNA and peptide binding. Then, the peptide chips were incubated with the fluorescently labeled RNA solutions for one hour at room temperature. After the incubation step, the peptide chip was washed with PBS-T (3 × 1 min) and briefly rinsed with deionized water (to wash out the rest of the phosphate buffer), dried with Argon, and stored.

### 2.5. Confocal Fluorescence Microscopy

After the incubation and washing steps, the peptide chips were scanned with a confocal fluorescent scanner Innoscan 1100 AL (Innopsys, Carbonne, France) at 635 nm and 532 nm with a resolution of 3 μm and a scanning velocity of 30 l/s. The PMT gain has been adjusted to maximize the contrast between the fluorescence signals and the background. Photometric tables for peptide libraries were made using Mapix software V9.1.0 (Innopsys, Carbonne, France). Fluorescent signals (Figure 1e,f) were used for comparative analysis of interactions between the RNAs and the cognate protocodes. The peptide spots with a higher fluorescence intensity correspond to a larger amount of labeled RNAs accumulated on the spots, i.e., higher fluorescence intensity corresponds to a stronger interaction between RNA and peptide. The same RNA concentration and the same incubation conditions make it possible to compare the intensities of fluorescent signals obtained both from different incubation wells and on different peptide chips.

### 2.6. Ranking of Binding Signatures

Using the fully combinatorial peptide libraries, we have developed a new method for analyzing combinatorial dynamics of amino acid signatures. This analysis consisted of calculating the interaction scores of all 3-mer, 4-mer, 5-mer, etc. peptide fragments until all possible fragments are evaluated. Peptide fragments were compiled based on peptide libraries. For example, a peptide AECD was represented as a set of the following fragments: {A, E, C, D, AE, EC, CD, AEC, ECD, AECD}. For the dominant AU-protocode, 135 unique 3-mers, 633 unique 4-mers, and 3131 5-mers were obtained. The number of 6-mers corresponded to the number of unique peptides in the AU peptide library. For the dominant GC-protocode, 74 unique 3-mers, 264 unique 4-mers, 1030 unique 5-mers, and 4100 unique 6-mers were obtained. The number of 7-mers corresponded to the number of unique peptides in the GC peptide library. Each peptide fragment m was associated with a set of fluorescent signals I_m_ from peptides containing this fragment. The interaction score R_m_ of peptide fragments was calculated as the average fluorescence intensity of all the peptides where this fragment occurs, divided by the corresponding standard deviation:

R_m_ = Mean (I_m_)/Standard Deviation (I_m_). (1)

Thus, the interaction score Rm increases if the mean fluorescent signal of the pep-tides with fragment m increases. Rm decreases if the standard deviation of the fluorescent signals from the peptides with fragment m increases. The algorithm was implemented using Python.

This section may be divided by subheadings. It should provide a concise and precise description of the experimental results, their interpretation, as well as the experimental conclusions that can be drawn.

## 3. Results

### 3.1. RNA Interactions with the AU Peptide Library

Table 1 enables the comparison of the means and the standard deviations of the fluorescent intensity signals of the AU-protocode peptides after their incubation with the RNA. The first feature of this comparison is that the dominant AU-protocode has a much stronger binding with the 12-mer adenine RNA compared to the recessive protocode: the fluorescence intensity ranges of both protocodes, determined by standard deviations, do not overlap. The second feature is that the binding of both protocodes with 12-mer uracil RNA is two orders of magnitude lower than with 12-mer adenine RNA.

**Figure 2:**
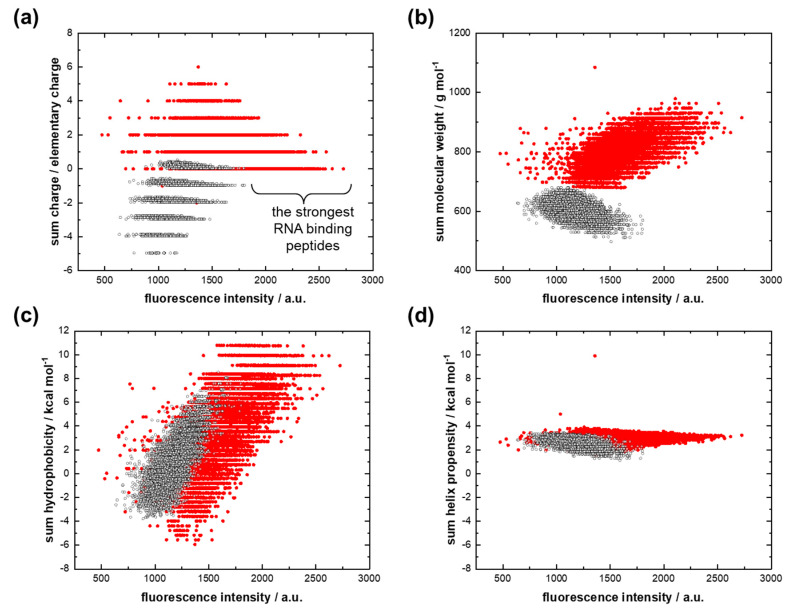
Interactions of the dominant AU library (red dots) and the recessive AU library (black dots) with 12-mer adenine RNA. (**a**) Fluorescence intensity versus the sum charge; (**b**) fluorescence intensity versus the sum molecular weight; (**c**) fluorescence intensity versus the sum hydrophobicity; (**d**) fluorescence intensity versus the sum helix propensity.

The reasons for the first feature can be explored using scatter plots, where the property of each peptide as sum charge [44], sum molecular weight, sum hydrophobicity [45], and sum helix propensity [46] are plotted against fluorescence intensity (Figure 2). Figure 2a shows a tendency toward a decrease in the sum charge as the fluorescent signal increases for both AU-protocodes. The peptides of the dominant AU-protocode, which bind more strongly to the 12mer adenine RNA, have a higher molecular weight, as well as a higher sum hydrophobicity (Figure 2b,c). The sum helix propensity did not significantly affect the strength of the interaction. Its magnitude converged to about 3 kcal/mol.

As noted above, the interaction of both AU-protocodes with 12mer uracil RNA (Figure S1a–d, Supplementary Materials) is significantly weaker than the interaction of AU-protocodes with 12mer adenine RNA. At the level of these weak interactions, there is a trend toward an increase in fluorescence intensity with an increase in the sum charge of the peptides, regardless of the sign of the charge for both the dominant and recessive AU-protocode (Figure S1b, Supplementary Materials).

**Figure 3:**
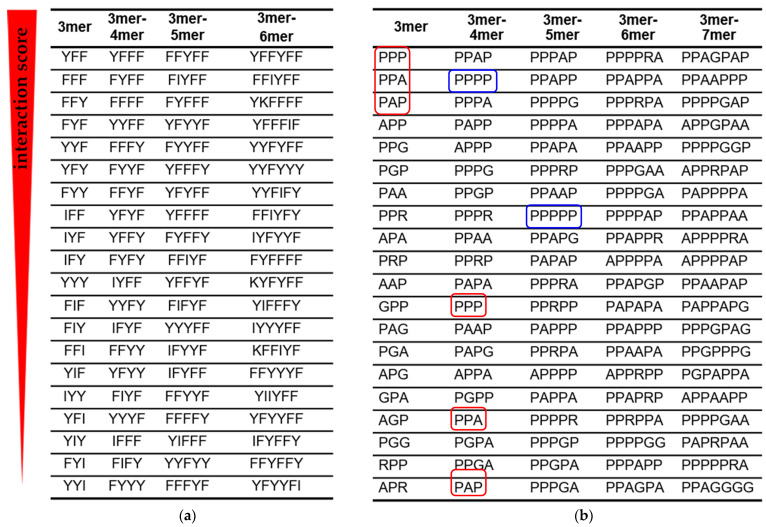
Amino acid signatures of RNA–peptide interactions. Peptide fragments with the highest interaction score are arranged in descending order (top to bottom). (**a**) Peptide fragments from the AU peptide library when interacting with 12-mer adenine RNA. (**b**) Peptide fragments from the GC peptide library when interacting with 12-mer cytosine RNA. Shorter fragments (red frames) are displaced by longer fragments from the list of signatures with the highest interaction score. The blue frames show the displacement of polyprolines from the position of the strongest binders to 12-mer cytosine RNA as the fragment length increases.

Using interaction scores, we grouped the peptide fragments into columns so that the previous column has all (3,…, n)-mer fragments, and the next (3,..., n + 1)-mer fragments (Figure 3). Such alignment allows an assessment of how the strongest RNA–peptide interactions change with increasing fragment length and amino acid composition.

Two amino acids with aromatic side chains Phe (F) and Tyr (Y) dominated the amino acid signatures of the dominant AU-protocode. As the peptide length increased, short binding signatures were displaced by the longer ones (Figure 3a). With an increase in the length of the signature, an increase in the number of Phe and Tyr amino acids was observed. These results strongly correlate with previous observations via aptamer technology that the A-rich RNA active site strongly favors the aromatic side chains of Phe and Tyr [12,47]. At the same time, other amino acids of the dominant AU-protocode, Ile (I) and Lys (K) were emerging among the highest interaction-ranking signatures and have begun to displace signatures composed solely of Phe and Tyr.

### 3.2. RNA Interactions with the GC Peptide Library

By analogy with Table 1, we present Table 2 for the mean values and standard deviations of fluorescent signals in the dominant and recessive GC-protocodes. The dominant GC-protocode has a significant binding with 12-mer cytosine RNA compared to the recessive GC-protocode: the fluorescence intensity ranges of both protocodes, determined by standard deviations, do not overlap. The average fluorescence intensity of both GC-protocodes in the case of interaction with 12-mer guanine RNA is 2–3 times lower than in the case of interaction with 12-mer cytosine RNA. Against this background, the extremely weak interaction of 12-mer uracil RNA with AU-protocodes is noteworthy (cf. Table 1). This may be explained by the fact that uracil does not have amino groups, unlike adenine, guanine, and cytosine.

**Figure 4:**
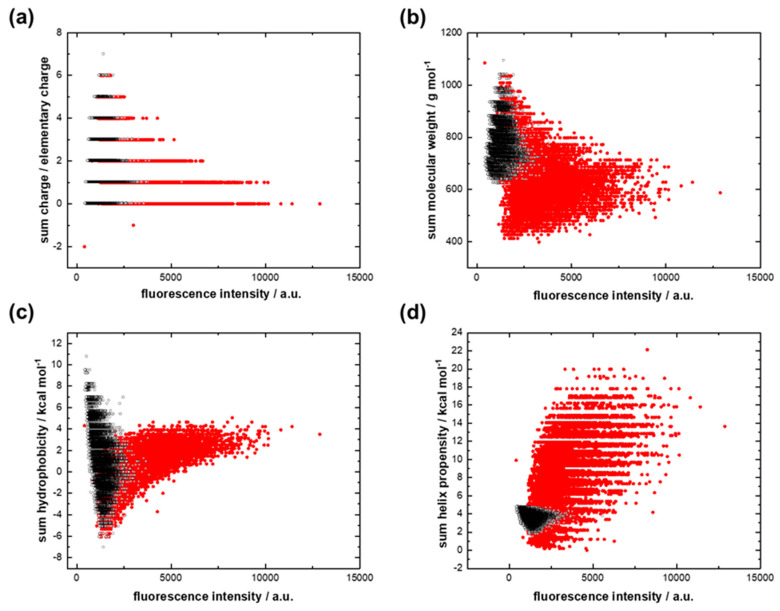
Interactions of the dominant GC library (red dots) and the recessive GC library (black dots) with 12-mer cytosine RNA. (**a**) Fluorescence intensity versus the sum charge; (**b**) fluorescence intensity versus the sum molecular weight; (**c**) fluorescence intensity versus the sum hydrophobicity; (**d**) fluorescence intensity versus the sum helix propensity.

The binding strength of the dominant GC-protocode with 12-mer cytosine RNA increased as the sum charge of the peptides decreased (Figure 4a). The binding strength was not related to the molecular weight and the sum hydrophobicity of the peptides (Figure 4b) but increased largely with an increase in the sum helix propensity (Figure 4d). Analogous plots for the case of interaction of both GC-protocodes with 12-mer guanine RNA are shown in Figure S2 (Supplementary Materials).

According to the interaction scoring (Figure 3b), the small hydrophobic amino acid Pro (P) plays a decisive role in the interaction with 12-mer cytosine RNA (blue frames in Figure 3b). Polyprolines have exhausted the potential of strong binders at the level of 5-mers. The stronger 5-mer signatures already contained other amino acids of the dominant GC-protocode such as Ala (A), Gly (G), or Arg (R). The PPA and PAP triplets with the highest interaction scores were displaced from the top positions as the length of the peptide fragments in the “3mer-4mer” column increased (red frames in Figure 3b).

**Figure 5:**
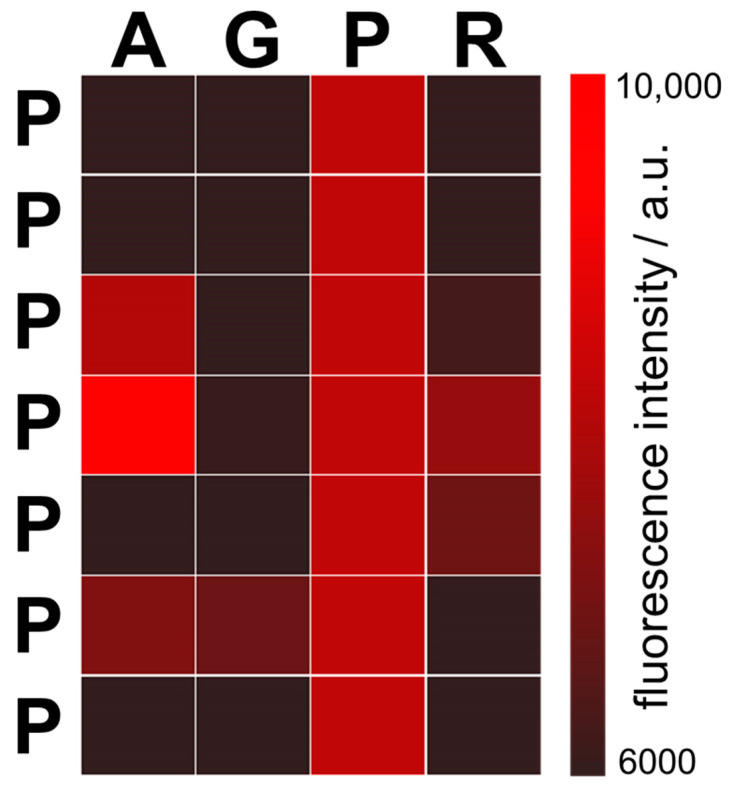
Fluorescent signals from the peptides after substitutions of 7-mer polyproline PPPPPPP. Each position in the polyproline was substituted with amino acids Ala (A), Gly (G), or Arg (R) from the dominant GC-protocode. For example, the upper left square represents the peptide APPPPPP, and the bottom right square the peptide PPPPPPR. All modified peptides were incubated with the 12-mer cytosine RNA. The peptide PPPAPPP (4th position in the first column with the highest fluorescence intensity) binds the 12-mer cytosine RNA most strongly.

In the first approximation, stronger binders can be considered as combinations of amino acid triplets. For example, the peptide PPAGPAP containing the PPA and PAP triplets had the highest interaction score in the dominant GC-protocode. The PPA and PAP triplets in PPAGPAP are separated by Gly, which provided them with a more flexible mutual orientation. Note that PPA and PAP triplets were inferior to the PPP triplet in terms of interaction score in the “3mer” column (Figure 3b). In substitution analysis of 7-mer polyproline, the peptide PPPAPPP revealed the strongest binding with the 12-mer cytosine RNA (Figure 5).

According to Figure 4d, the increased accumulation of 12-mer cytosine RNA on peptide spots positively correlates with the sum helix propensity of the growing chains. The dominant GC-protocode could be considered as a donor of standard secondary structures after the combinatorial fusion of AU- and GC-protocodes. Kubyshkin and Budisa pointed out the importance of the amino acids Gly, Ala, and Pro in the formation of the α-helix as the core chemical scaffold for the evolution of proteins [48]. Hartman and Smith concluded that several early structural types would be produced by the early GC code, coding for Gly, Ala, Pro, and Diapr (hypothesized non-canonical amino acid diamino proprionic acid instead of Arg) [49].

## 4. Discussion

### 4.1. The Combinatorial Fusion Cascade

Hartman and Smith also proposed the gradual expansion of the coding space as GC–GCA–GCAU genetic code [49]. This hypothesis is supported by the late branching of Class I aaRS (aminoacyl-tRNA synthetases) compared to the Class II aaRS [50,51], which includes most amino acids of the GC-protocodes Gly, Ala, Pro, Thr, and Ser. It is assumed that the entry of A and U into the replication molecular apparatus of the GC phase expanded the number of codons that were initially occupied by smaller, more stable amino acids and then redistributed as new amino acids emerged during the coevolution of proteins and RNA [52].

But what could be the molecular apparatus around the complementary bases A and U before they entered the GC phase? Since the discovery of the genetic code, a remarkable relationship between the hydrophobicity of the amino acids and the second base of its codon has been noted. All codons having U as the second base are associated with the most hydrophobic amino acids, and those having A as the second base are associated with the most hydrophilic amino acids [53,54]. This property is natural for the recently proposed combinatorial fusion cascade (CFC), a hypothetical autocatalytic reaction leading to the formation of the SGC (Figure S3) [55]. According to the CFC, such a division of amino acids was the first stage in the formation of the genetic code before the appearance of the protocodes, at the level of competing amino acid pairs associated with complementary homo-ribonucleotides. The formation of homo-ribonucleotides of adenine and uracil could be preceded by the self-assembly of pure adenosine and pure uridine monophosphate strands on Montmorillonite clay, experimentally shown by Himbert et al. [56].

Dominant AU- and GC-protocodes, the fusion of which to the SGC is described by the Watson–Crick mutations, have strong similarities. All hydrophobic amino acids of the protocodes are associated with triplets having the second base U or C, respectively. Both protocodes contain one positively charged amino acid Lys or Arg. Finally, both protocodes contain amino acids responsible for the strongest bonds with cognate homo-ribonucleotides, which we have shown experimentally. Such similarity may suggest the existence of early forms of multiplicators based on the AU- and GC-protocodes, as necessary elements for the emergence of a living cell.

The CFC hypothesis has unexpected implications, emphasizing the importance of hydrophobic amino acids:

1. Small amphiphilic amino acid–RNA complexes based on complementary RNAs played an important role in primordial translation. Hydrophobicity was provided by hydrophobic amino acids;
2. Each hydrophobic amino acid directly determined its hydrophilic partner amino acid.

The second property could be achieved if amphiphilic complexes formed membranes with adjustable stereochemical features, for example, due to the specific distance between two amphiphilic complexes within the membrane. Such catalytic membranes are currently unknown, but they may have significant advantages at an early stage in the development of replicators. In particular, the well-known problem of precursor transport and high metal ion concentration required for non-enzymatic replication within lipid vesicles would be bypassed [57]. Such membranes could form compartments with purely structural functions for their movement and accumulation of products of the intramembrane synthesis inside them.

### 4.2. The Standard Genetic Code as a Message

Since the origin of the genetic code is still at the stage of hypotheses, it would be justified to look for different approaches to it. In this work, we have applied the “true message” approach to the code partition. The codon assignment of the amino acids is a unique true message from the last universal common ancestor named LUCA. On the other hand, we can only make assumptions about the physicochemical conditions for the SGC emergence on early Earth. According to the “true message” approach, the origin of the code is considered directly from the SGC itself by identifying the most universal rules that could lead to its formulation. The identification of the combinatorial fusion rules of protocodes enabled the formulation of the combinatorial fusion cascade hypothesis discussed in Section 4.1.

An alternative to the “true message” approach is the “out of chaos” approach. It consists in choosing a primitive code based on some physicochemical properties of a reduced set of amino acids. An example of such an approach is the GADV hypothesis of Ikehara [58]. The GADV hypothesis identifies a primitive code based on the four amino acids Gly, Ala, Asp, and Val due to their properties of forming secondary structures such as turn/coil, alfa-helix, and beta-sheet. The “out of chaos” approach can be very powerful if the chosen physicochemical properties determine the origin of the genetic code. However, the “out of chaos” approach is associated with the experimentally unverified assumptions about the existence of the primordial soup and the principles of codon reassignments as new amino acids entered the code. It is worth noting that one hypothesis cannot be used as an argument against another at those points where the “true message” approach contradicts the “out of chaos” approach. In particular, the argument about random polymerization or random interactions cannot be applied, since the optimal conditions for the presence of monomers in equal concentrations on early Earth have not been experimentally confirmed.

The stereochemical hypothesis refers to the “true message” approach, as it proceeds from an experimental search for selective bonds between amino acids and their cognate codons from the SGC. In the introduction, we referred to the criticism of the stereochemical hypothesis. In our experiments, the fact of strong interactions of Phe with the 12-mer adenine RNA and Pro with the 12-mer cytosine RNA can not be interpreted in favor of the selective bonds of the amino acids with their cognate triplets. As noted in the introduction, Polyansky and Zagrovich provide a new perspective on the interpretation of the stereochemical hypothesis, suggesting that codon distribution occurred after the formation of primordial RNA–peptide complexes, which could subsequently provide selective binding of individual amino acids [22]. The search for such selective RNA–peptide complexes could be carried out using high-density peptide arrays.

### 4.3. Peptide Arrays for Screening Binders to Ancient Folded RNAs

As noted above, the results on the strong binding of phenylalanine and tyrosine to homo-oligonucleotides of adenine, which we obtained using a linear RNA, correlated with experiments where A-rich RNA fragments were located on the hairpin loop as part of a more complex folded RNA [12,47]. Recalling that phenylalanine and tyrosine are encoded in the dominant AU-protocode by the UUU and UAU triplets, respectively, the strong interactions of these amino acids with peptides can be interpreted as strong interactions of Phe and Tyr with their anticodons. Strong enrichment of Phe near its anticodons was noted when analyzing the spatial distribution of amino acids relative to rRNA in ribosomes from four species (one archaebacterium and three eubacteria) [16]. Such enrichment was outside the statistical null hypothesis significance with a p-value of 0.012. The same trend was noted by Polyansky and Zagrovic when studying interface statistics for adenine (A) in interactions between messenger RNAs and their cognate proteins. In particular, the A-preference of amino acids correlated inversely with the A-content (R = 0.59) [22]. Thus, high-density peptide arrays have a great potential for identifying specific binders to individual single-stranded elements within more complex RNA structures.

Jaeyoung Pai et al. incubated six hairpin RNAs directly onto peptide arrays containing 111 peptides [59]. Although the number of peptides was significantly limited compared to those used in this work, the authors showed the possibility of finding the inhibitory activity of RNA-binding peptides in cells. There are several scenarios for the formation of tRNAs from ancient hairpin RNAs [60,61,62]. Considering that α-helical peptides containing both natural and unnatural amino acids are reasonable ligands for hairpin RNAs [63,64,65], the high-density peptide arrays may become one of the important experimental methods for studying the primordial co-evolution of ribonucleic acids and peptides.

## 5. Conclusions

High-density peptide arrays were used for the first time to study primordial interactions between RNA and peptides under physiological conditions. Canonical amino acids were partitioned into dominant and recessive GC- and AU-protocodes competing for the same codons. This partition absorbed the ideas of the uniqueness of the genetic code, as well as the results of bioinformatic studies of RNA–peptide complexes, according to which there was a separate phase in the development of the genetic code with RNA containing guanine and cytosine, expanded later by adenine and uracil. The features of the interaction of peptides from the protocodes with cognate 12-mer homo-oligonucleotides of adenine, uracil, guanine, and cytosine were studied.

The synthesized peptide libraries contained all combinatorial combinations of 6-mer peptides from the AU-protocodes and 7-mers of peptides from the GC-protocodes. The amino acids methionine and tryptophan were not considered, since they entered the genetic code at the last stages of its optimization. Full combinatorial libraries allowed a comprehensive analysis of the strongest peptide binders, down to the effect of individual amino acids in the peptide on interaction with RNA. In addition, a new method for evaluating molecular interactions based on the calculation of interaction scores for individual fragments of peptides was presented.

In the AU protocodes, the strongest binders to the 12-mer of adenine RNA were composed of two amino acids from the dominant AU-protocode: phenylalanine and tyrosine. The average binding strength of the AU-protocodes with 12-mer uracil RNA was two orders of magnitude lower than with 12-mer adenine RNA and significantly lower than the average binding strength of the GC-protocodes with their cognate 12-mer homo-oligonucleotides. In the dominant GC-protocode, the amino acid proline plays an exceptional role, providing the strongest binding to the 12-mer cytosine RNA. However, polyprolines have exhausted their binding potential at the 5-mer level. As a first approximation, strong binders to 12-mer cytosine RNA can be considered as combinations of amino acid triplets with the highest interaction scores. For both dominant AU- and GC-protocodes, RNA binding increased with increasing peptide length.

This paper discusses the consequences of partitioning based on protocodes from the point of view of the combinatorial fusion cascade hypothesis.

We believe that high-density peptide arrays are a powerful tool not only for studying the interaction of RNA with short peptides but also for engineering primordial-like RNA–peptide complexes with new catalytic and selective functions.

## Figures and Tables

**Table 1:** The mean and the standard deviation of the fluorescent intensity signals (arb. units) of the AU-protocode peptides after their incubation with the RNA.

Protocode RNA	AU Dominant 12-mer A	AU Recessive 12-mer A	AU Dominant 12-mer U	AU Recessive 12-mer U
Mean	1569	1191	56	61
Stand. deviation	228	148	8	14

**Table 2:** The mean and the standard deviation of the fluorescent intensity signals (arb. units) of the GC-protocode peptides after their incubation with the RNA.

Protocode RNA	GC Dominant 12-mer G	GC Recessive 12-mer G	GC Dominant 12-mer C	GC Recessive 12-mer C
Mean	684	559	3117	1248
Stand. deviation	251	196	1299	433

## Data Availability

Tables with fluorescent intensity values for each peptide from libraries synthesized on peptide chips, as well as calculated values of the sum charge, the molecular weight, the sum hydrophobicity, and the sum helix propensity are available in the Zenodo repository, https://doi.org/10.5281/zenodo.7594469 (accessed on 1 March 2023).

## Supplementary Materials

The following supporting information can be downloaded at: https://www.mdpi.com/article/10.3390/life13030796/s1, Figure S1: Interactions of the dominant AU-protocode (red dots) and the recessive AU-protocode (black dots) with 12-mer uracil RNA. (a) Fluorescence intensity versus the sum charge; (b) fluorescence intensity versus the sum molecular weight; (c) fluorescence intensity versus the sum hydrophobicity; (d) fluorescence intensity versus the sum helix propensity. Figure S2: Interactions of the dominant GC-protocode (red dots) and the recessive GC-protocode (black dots) with 12-mer guanine RNA. (a) Fluorescence intensity versus the sum charge; (b) fluorescence intensity versus the sum molecular weight; (c) fluorescence intensity versus the sum hydrophobicity; (d) fluorescence intensity versus the sum helix propensity. Figure S3: The combinatorial fusion cascade leading to the codon assignments in the SGC. The blue letters indicate the fusion rules for the dominant and recessive AAA/UUU- and GGG/CCC-pairs to the protocodes. The red letters indicate the fusion rules for dominant and recessive AU- and GC-protocodes to the SGC (see Section 2.1) [55].
