# Supplementary material for: Screening for Primordial RNA–Peptide Interactions Using High-Density Peptide Arrays
Source: Life (Basel). 2023 Mar 15;13(3):796. doi: 10.3390/life13030796 (PMC10053474; doi:10.3390/life13030796)
Supplement: Supplementary file 1 [file life-13-00796-s001.zip › life-2247129-supplementary.pdf]

life

Supporting Information

Screening for primordial RNA-peptide interactions using high-density peptide arrays

Felix Jenne , Ivan Berezkin , Frank Tempel , Dimitry Schmidt , Roman Popov ,

and Alexander Nesterov-Mueller

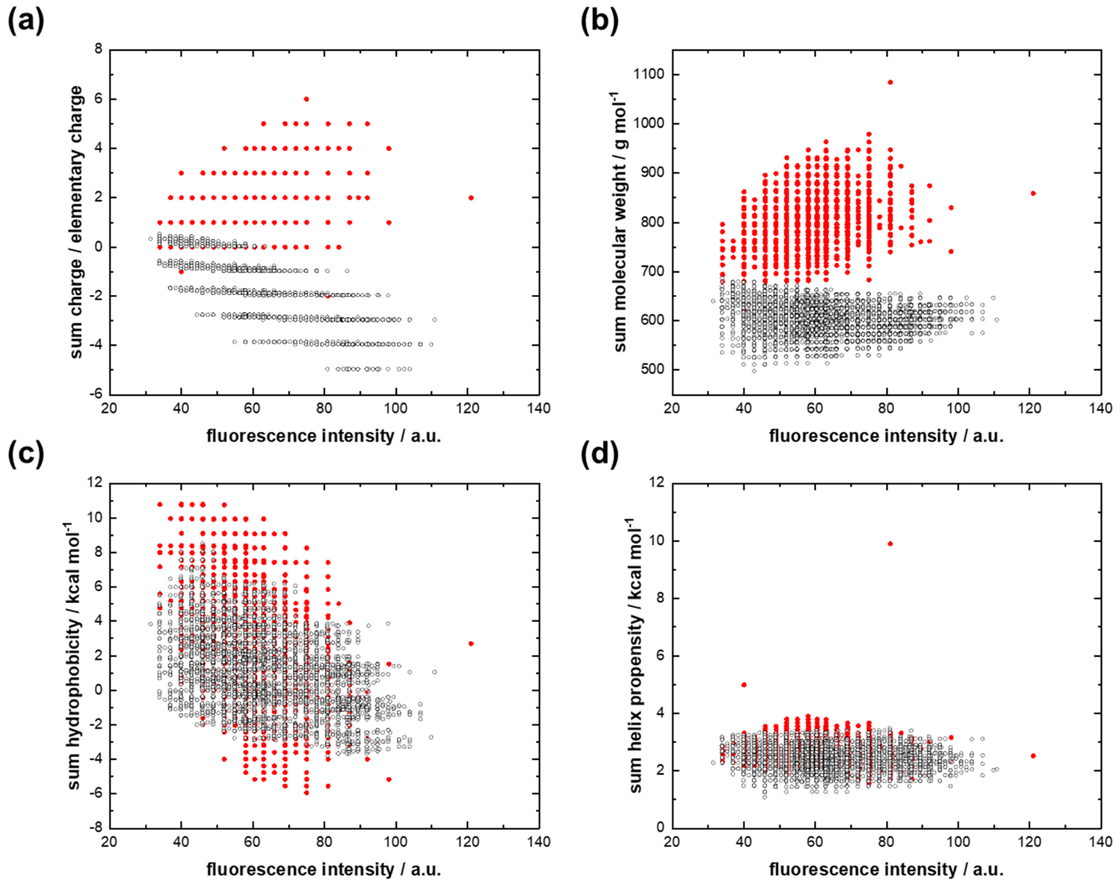

Figure S1. Interactions of the dominant AU-protocode (red dots) and the recessive AU-protocode (black dots) with 12-mer uracil RNA. **(a)** Fluorescence intensity versus the sum charge; **(b)** Fluorescence intensity versus the sum molecular weight; **(c)** Fluorescence intensity versus the sum hydrophobicity; **(d)** Fluorescence intensity versus the sum helix propensity.

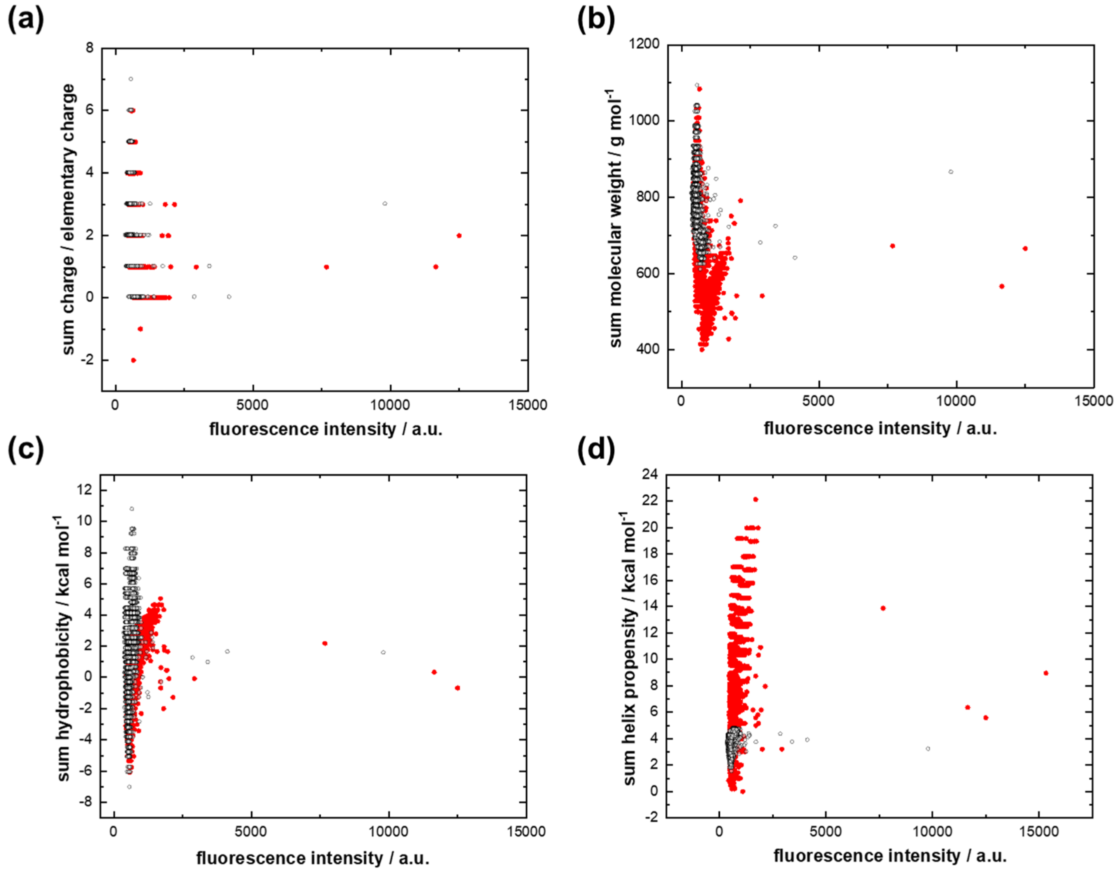

Figure S2. Interactions of the dominant GC-protocode (red dots) and the recessive GC-protocode (black dots) with 12-mer guanine RNA. **(a)** Fluorescence intensity versus the sum charge; **(b)** Fluorescence intensity versus the sum molecular weight; **(c)** Fluorescence intensity versus the sum hydrophobicity; **(d)** Fluorescence intensity versus the sum helix propensity.

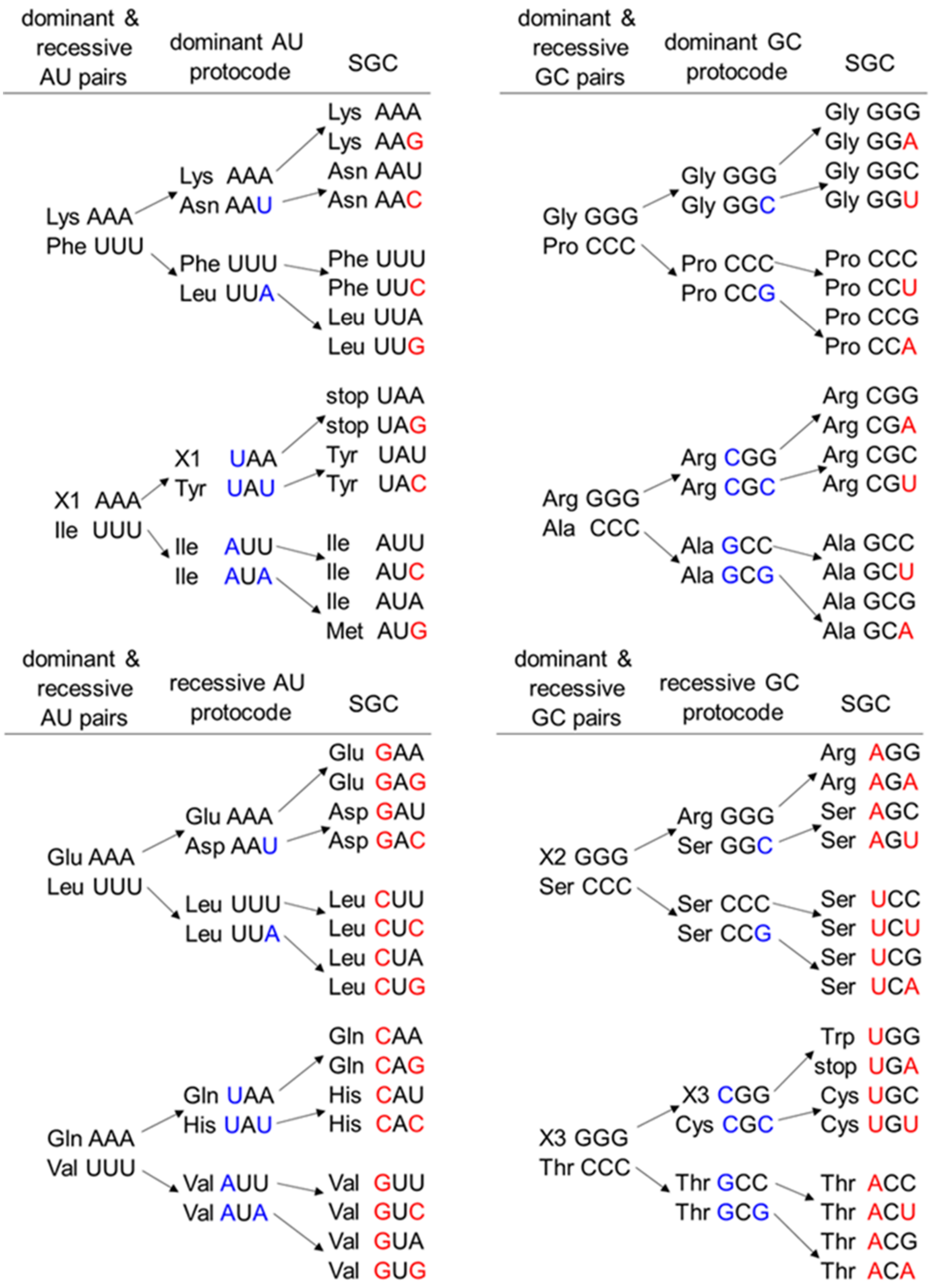

Figure S3. The combinatorial fusion cascade leading to the codon assignments in the SGC. The blue letters indicate the fusion rules for the dominant and recessive AAA/UUU- and GGG/CCC-pairs to the protocodes. The red letters indicate the fusion rules for dominant and recessive AU- and GC-protocodes to the SGC (see section 2.1) [55].
